# Oxidative Quality of Dairy Powders: Influencing Factors and Analysis

**DOI:** 10.3390/foods10102315

**Published:** 2021-09-29

**Authors:** Holly J. Clarke, William P. McCarthy, Maurice G. O’Sullivan, Joseph P. Kerry, Kieran N. Kilcawley

**Affiliations:** 1 Food Quality and Sensory Science Department, Teagasc Food Research Centre, Moorepark, Fermoy, P61 P996 Cork, Ireland; holly.clarke@teagasc.ie; 2 Sensory Science Group, School of Food and Nutritional Sciences, University College Cork, T12 R229 Cork, Ireland; maurice.osullivan@ucc.ie; 3 Food Chemistry and Technology Department, Teagasc Food Research Centre, Moorepark, Fermoy, P61 P996 Cork, Ireland; williamp.mccarthy@teagasc.ie; 4 Food Packaging Group, School of Food and Nutritional Sciences, University College Cork, T12 R229 Cork, Ireland; joe.kerry@ucc.ie

**Keywords:** lipid oxidation, dairy powder, sensory

## Abstract

Lipid oxidation (LO) is a primary cause of quality deterioration in fat-containing dairy powders and is often used as an estimation of a products shelf-life and consumer acceptability. The LO process produces numerous volatile organic compounds (VOC) including aldehydes, ketones and alcohols, which are known to contribute to the development of off-flavours in dairy powders. The main factors influencing the oxidative state of dairy powders and the various analytical techniques used to detect VOC as indicators of LO in dairy powders are outlined. As the ability to identify and quantify specific VOC associated with LO improves this review highlights how these techniques can be used in conjunction with olfactory and sensory analysis to better understand product specific LO processes with the aim of maximizing shelf-life without compromising quality.

## 1. Introduction

Oxidation of bovine milk fat is recognised as the main factor in the development of undesirable flavours in products such as whole milk powder (WMP) and infant milk formula (IMF). Lipid oxidation (LO) is responsible for the formation of primary and secondary oxidation products including aldehydes, ketones and alcohols, which can impact on nutritional and sensory properties of dairy powders [1]. Factors that can contribute to the oxidative stability of dairy powders include the quality of the raw milk (fatty acid (FA) composition, bovine diet, and storage conditions), processing parameters, powder composition (especially water activity), presence of pro- and anti-oxidants (natural or added during processing), packaging materials, storage and transport conditions. LO is a free radical chain reaction consisting of three stages; initiation, propagation, and termination. Free radicals and peroxides (tasteless, flavourless compounds) [2] are generated during the initiation phase when molecular oxygen reacts with unsaturated FA [3]. The rate of the propagation cycle is directly proportional to the degree of lipid unsaturation [4]. The resultant termination products (secondary LO products) are generally quite stable, however, it is these secondary LO products (mainly aldehydes, ketones, alcohols, and hydrocarbons) that actually contribute to off-flavour development and have been described as grassy, soapy, cardboard-like, painty, tallowy and/or fishy [5,6]. Secondary LO compounds can be monitored and quantified instrumentally as molecular-level indicators of oxidised flavours in dairy products. This measurement can be used in place or in combination with sensory analysis to provide an overall profile of the flavour stability of a dairy powder [6,7]. The presence and increase of numerous secondary LO products in dairy powders during processing and storage is well documented [6,8,9]. However, there is a lack of knowledge linking the quantification of volatiles associated with LO to descriptive sensory attributes in dairy products in general [10]. The aims of this review are as follows: (1) to summarize the main factors influencing the oxidation of dairy powders, (2) to summarise the various analytical techniques used to detect and quantify VOC as indicators of LO, and (3) to highlight the use of combined analytical and sensory approaches to better understand the LO process in dairy powders.

## 2. Bovine Milk Lipids

Bovine milk fat is one of the most complex fats found in nature and varies widely from animal to animal due to factors including dietary composition [11], breed [12], seasonality [13,14], and stage of lactation [15,16]. Milk fat contains over 400 different FA [17] which originate from diet [18], microbial activity in the rumen (and transported to the secretory cells via the blood and lymph), or from synthesis in the secretory cells. The main milk lipids are triglycerides comprised of a glycerol backbone with three esterified FA. The FA are composed of a hydrocarbon chain and a carboxyl group. The major FA found in milk are: C14:0—myristic (11% *w*/*v*), C16:0—palmitic (26% *w*/*v*), C18:0—stearic (10% *w*/*v*), C18:1—oleic (20% *w*/*v*, and short chain FA (11% *w*/*v*): C4:0—butyric, C6:0—caproic, C8:0—caprylic, and C10:0—capric [19]. A milk fat globule membrane (MFGM) is a surface-active membrane with a phospholipid structure that comprises of a polar lipid bilayer, proteins, enzymes, neutral lipids, and trace components, and envelops each fat globule [20]. Approximately 25% of the FA in milk are mono-unsaturated FA (MUFA) while 2.3% are poly-unsaturated with an omega-6/omega-3 ratio of around 2:3. Trans-FA comprise approximately 2.7% of total milk FA. The amount of poly-unsaturated FA (PUFA) consumed by ruminants is an important factor in the rate at which LO progresses because they are generally dehydrogenated in the rumen by microbial action and this impacts on subsequent levels in the milk. The most abundant FA in bovine milk is α-linolenic acid (C18:3) [21]. MUFA are not oxidised as readily as PUFA, however, the most abundant MUFA (oleic acid) in bovine milk is the source of important secondary oxidation products [10]. Saturated FA in milk are generally stable compounds that are not easily oxidised and thus are not major LO contributors in dairy powders. The abundance of individual FA in milk and milk products is important [22] as they can dictate the rate at which LO progresses, but also which specific oxidation products are formed.

## 3. Mechanism of Lipid Oxidation

It is generally accepted that oxygen reacts naturally with many organic substrates resulting in the formation of primary oxidation products; hydroperoxides and other oxygenated compounds. There are three known types of LO that can affect dairy products; auto-oxidation, photo-oxidation, and metal induced oxidation [23].

**Figure 1:**
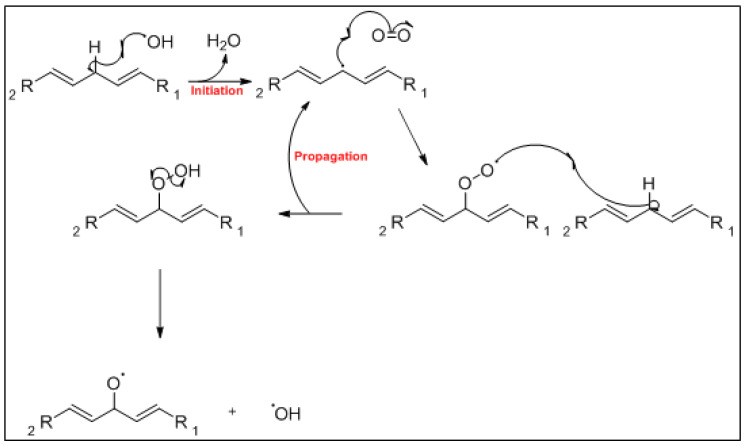
The mechanism of the first two phases of the lipid oxidation process; initiation and propagation.

**Figure 2:**
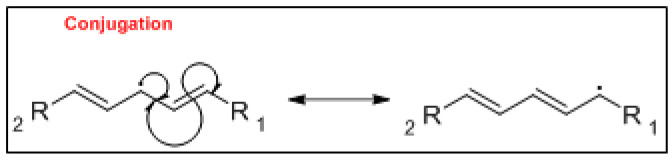
The mechanism of conjugation of the lipid oxidation process.

**Figure 3:**
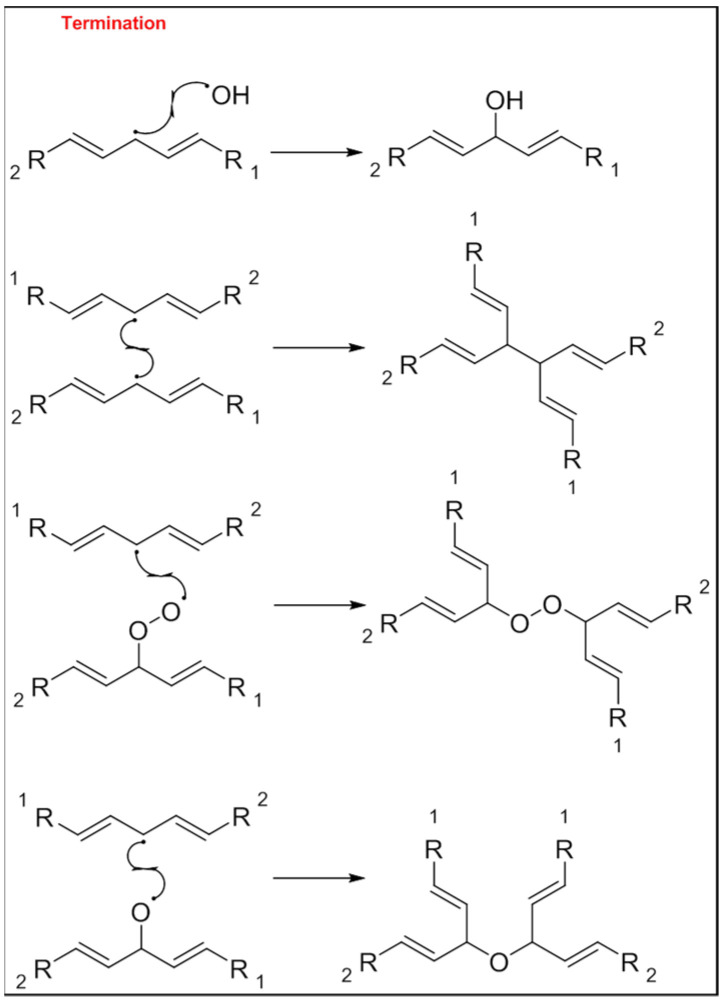
Common termination products of the lipid oxidation process.

The mechanism of the auto-oxidation of PUFA as a radical chain reaction was established more than half a century ago. The process of LO can be broken into three distinct, but partially overlapping phases of radical reactions; initiation, propagation and termination [24] (Figure 1, Figure 2 and Figure 3). Free radicals and peroxides, both of which are highly reactive, are generated during the initiation phase when molecular oxygen reacts with unsaturated FA. In addition to oxygen, oxidative initiators such as chemical oxidisers, transition metals (e.g., copper and iron), and enzymes (e.g., lipoxygenases) contribute to the rate of the initiation phase [3]. Heat and light also exacerbate the rate of the initiation phase and the other phases of LO [25]. The rate of auto-oxidation is increased by increasing unsaturation of the alkyl chain [25], and the matrix also plays a role in the susceptibility of a product to oxidation [26]. FA alkyl chains are susceptible to oxidation at alkene bonds and neighbouring allylic carbons.

Photo-oxidation and free-radical reactions at allylic carbons are responsible for the breakdown of unsaturated lipids [24,25,27]. These reactions produce hydroperoxides in these allylic bonds, and cause changes in the position and geometry of double bonds. Auto-oxidation and photo-oxidation are associated with different hydroperoxide reaction products, indicating that different reaction mechanisms are involved [28]. Photo-oxidation of milk has been well documented [23,29], exposure to light, either natural or artificial, can cause development of off-flavours in milk within 15 min [30]. The subsequent aromas have been characterised as burnt protein, cabbage-like and plastic [23], however, their intensity can decrease the longer the milk is exposed to light, allowing newly activated off-flavours to dominate. These off-flavours have been described as cardboard-like, metallic and rancid [31,32,33]. Exposure to ultra-violet (UV) light can enable the oxidation of fat to volatile aldehyde compounds and has also been found to cause the degradation of sulfur–containing compounds, both of which are major contributors to off-flavours in milk [30]. A study by Silcock et al. [34] reported good correlation between negative sensory perceptions and VOC formation for milk stored in light-exposed containers, these include photo-oxidation and auto-oxidation compounds such as dimethyl disulfide, and aldehydes such as heptanal, pentanal and hexanal. For milk stored in containers protected from light, no correlation between the sensory attributes and VOC was documented.

Furthermore, the type of light the product is exposed too can also have an impact on the levels of oxidation. A study by Brothersen et al. [30] demonstrated that exposure of milk to fluorescent light (commonly used in the retail of dairy products) resulted in greater changes in LO levels, compared with exposure to white light-emitting diodes (LED). This study demonstrated that even high quality milk is susceptible to photo-oxidation at the point of sale dependent upon the type of lighting.

**Figure 4:**
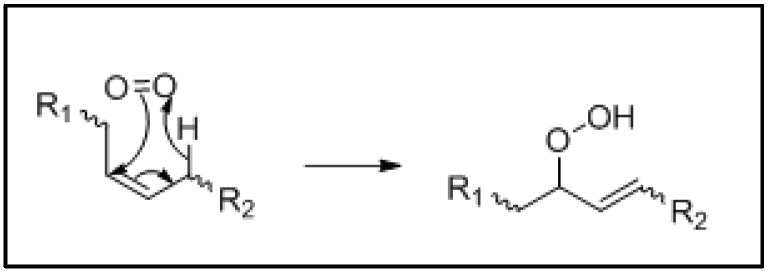
Ene reaction of an olefinic bond with a singlet oxygen. The formation of the hydroperoxide can happen at either of the olefinic sp2 hybridised carbons.

There are two mechanisms by which photo-oxidation may occur; (1) a radical cascade reaction initiated by the removal of a hydrogen or electron from an unsaturated allylic FA system, or (2) when oxygen is converted to its excited singlet state and reacts rapidly with an olefinic bond producing hydroperoxides on one of the original olefinic carbons and shifting of the cis bond to a trans configuration (Figure 4).

## 4. Secondary Reactions Associated with Lipid Oxidation

**Figure 5:**
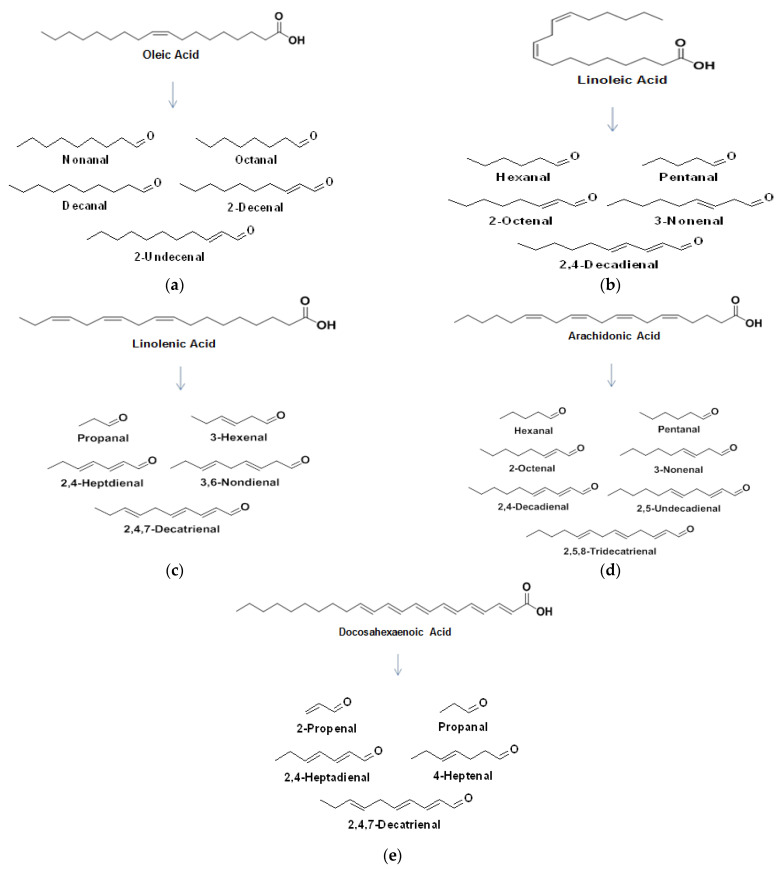
Some major fatty acids found in milk and some of their associated breakdown products; (**a**) oleic acid, (**b**) linoleic acid, (**c**) linolenic acid, (**d**) arachidonic acid, and (**e**) docosahexaenoic acid.

Various FA within milk are broken down via oxidation to primary and secondary oxidation products. The formation of a hydroxyperoxide through the oxidative mechanisms discussed earlier, breaks down to form an alkoxyl radical which splits by homolytic β-scission each side of the carbon bonded to the oxygen radical. The major FA in milk and some of their associated breakdown products are outlined in Figure 5a–e.

### 4.1. The Maillard Reaction

**Figure 6:**
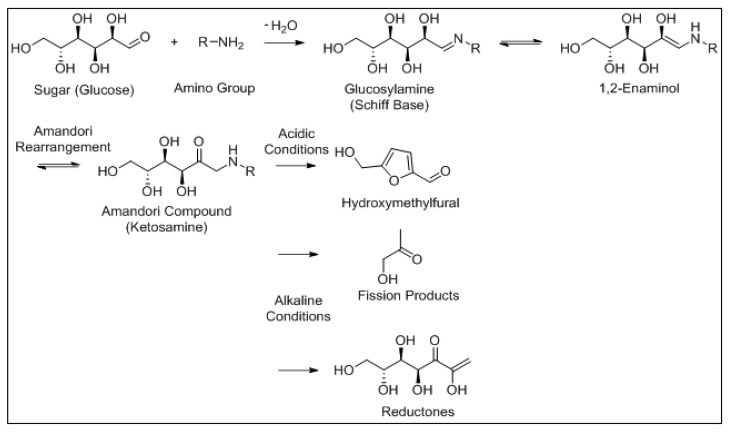
Schematic of the Maillard reaction of glucose with a generic amino group RNH2. The carbonyl functional group on the sugar undergoes a substitution reaction with the amino group of a protein or amino acid to form an N-substituted glycosylamine. This undergoes isomerisation by undergoing an Amandori rearrangement forming a ketosamine. This can undergo a number of reactions to produce a range of compounds which can undergo further reactions.

Along with LO, the Maillard reaction is an important chemical reaction that occurs in numerous foods, and both reactions have been shown to influence each other [35]. The Maillard reaction is a well-documented, non-enzymatic browning reaction between the amine groups of free amino acids, peptides or proteins and reactive carbonyl groups of reducing sugars under thermal processing and/or storage conditions [36]. This reaction can occur at room temperature, but is optimal at much higher temperatures (140–165 °C). The Maillard reaction has been identified as a main factor in quality deterioration of IMF [37]. However, in whey protein concentrate (WPC) and whey protein isolate (WPI), Maillard reaction products contribute to a lesser extent to flavour formation than LO [38,39]. The moisture content must be below 3% *w*/*w* for the Maillard reaction to conclude, a value that is not reached in most dried dairy products [40]. The Maillard reaction mechanism is outlined in Figure 6.

### 4.2. The Strecker Reaction

**Figure 7:**
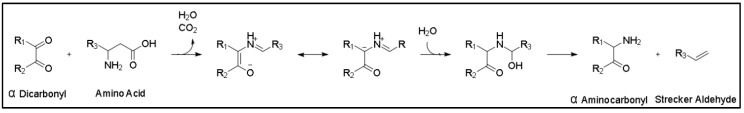
Schematic of the Strecker degradation mechanism, where an α-dicarbonyl compound and an amino acid undergo a decarboxylation reaction to form an α-aminocarbonyl compound and Strecker aldehyde product.

Similar to the Maillard reaction, the Strecker reaction mechanism is also linked to LO. Aldehydes are readily converted to secondary alcohols or acids and are therefore known as transitory volatile compounds with some known to be a result of Strecker reactions [41,42]. The degradation of amino acids during the Strecker reaction is one of the primary mechanisms resulting in the final aroma compounds of the Maillard reaction. The process involves the oxidative deamination and decarboxylation of the amino acid in the presence of α-dicarbonyl compounds formed in the Maillard reaction and the formation of the corresponding Strecker aldehyde [43,44]. Each amino acid produces a specific Strecker aldehyde which comprises one carbon atom less than the amino acid from which it is formed. Strecker aldehydes such as 3-methylbutanal (malty flavour) [45] and phenylacetaldehyde (honey-like flavour) are derived from leucine and phenylalanine, respectively, and are commonly reported as aroma contributors in dairy products [46]. LO and Maillard reactions interact in complex food systems and can share common chemical mechanisms and intermediate compounds [35]. Moreover, certain carbonyls derived from LO such as alkadienals and ketodienes have been shown to promote the oxidative degradation of amino acids to produce the corresponding Strecker aldehydes via Strecker-type reactions [47,48]. The Strecker reaction is outlined in Figure 7.

## 5. Lipid Oxidation in Dairy Powders

### 5.1. Whole Milk Powder

Due to its high fat content (26–42% *w*/*w*) and significant amount of exposed fat on its surface, WMP [6] is highly susceptible to LO during processing, transport and storage, which can adversely impact its sensory and nutritional properties. Some odour-active compounds have already been identified in WMP [49], with the most important off-flavour compounds resulting from LO such as, hexanal, other aldehydes and ketones. Determining the cause of undesirable LO changes in WMP flavour is complex owing to the fact that many of the aroma-active compounds are produced by two or more mechanisms [6,49,50]. Moreover, differences in the FA profile of WMP influences its susceptibility to LO [51,52]. Bovine feeding system is one of the major factors affecting the FA profile of milk and milk powders [11,53]. Maintaining quality and potentially increasing the storage stability and shelf-life of WMP is of great importance to manufacturers. Whetstine and Drake [49] documented changes in the flavour of WMP at ambient temperatures and found that the formation of off-flavours occurred after 3 months of storage, primarily as a result of LO. It is also important to note that off-flavours in dairy powders can carry over into product applications [49], therefore having information in relation to the LO status of the starting powder is very important not only for the oxidative stability of the powder itself but also for future applications.

### 5.2. Skim Milk Powder

Few studies have focused on the impact of LO on the quality and stability of SMP, likely due to its low fat content (0.6–1.25% *w*/*w*). SMP should have a flavour similar to that of fluid milk [54]; however, differences in manufacturing processes [55,56] and milk composition [57] can result in the formation of different flavour characteristics and intensities. Shiratsuchi et al. [58] was one of the first studies to profile the VOC content and flavour of SMP. The compounds identified included aldehydes, ketones, alcohols, esters, furans and, phenolic compounds, and was also one of the first studies to identify monoterpene and sesquiterpene hydrocarbons in milk. Methyl ketones were abundant in SMP, but were below their flavour thresholds. Alcohols were also found to have little influence on SMP flavour. The primary contributors to the flavour of SMP were free FA, comprising approximately 79% of the total VOC profile, however lactones were also present at high concentrations.

Abdalla et al. [57] evaluated the sensory characteristics of nonfat dry milk (NFDM) and SMP, and found that the intensity of the heat treatment used during production influenced the flavour of the final product, with medium heat powders having a cooked flavour, and low heat powders having oxidised and metallic flavours. Heat treatment can exacerbate the intensities of undesirable flavours in SMP. Whetstine and Drake [49] found that the impact of LO on the flavour of SMP was much more variable than with WMP. The authors also found that some SMP developed off-flavours immediately upon storage, while the flavour of others remained stable throughout storage. These results are somewhat surprising as it was anticipated that SMP should be less susceptible to LO than WMP due to its much lower fat content. However, the fat in WMP and in other high-fat dairy powders may act as a solvent for secondary oxidation products, especially non-polar molecules, impacting their transition to the gaseous phase. Thus, the low fat content in SMP may result in non-polar oxidative products being more easily perceived [59], as in theory they should be more easily transferred from the fat phase to the gaseous phase (or to the aqueous phase when hydrated).

### 5.3. Infant Milk Formula

The fat content of IMF is approximately 28% *w*/*w* and is designed to contain a FA composition similar to that of human milk. This is generally attained through the addition of fish, soya and/or vegetable oils [60]. Increased levels of PUFA from these sources may however result in an unstable product that is highly susceptible to LO [10]. For this reason, understanding the modifications of PUFA in IMF is important with regard to the stability and safety of IMF throughout its proposed shelf-life. A review by Saphier and Silberstein [61] focused on the storage conditions of IMF and the levels of LO. The study concluded that IMF comprising more unsaturated FA was more susceptible to LO, and that exposing IMF to known LO contributors (oxygen and elevated temperatures of >37 °C) increased the rate of LO.

A study by Romeu-Nadal et al. [7] focused on the oxidative stability of milk formulas (packed in sealed aluminium foil bags flushed with N_2_) that had been supplemented with various FA and stored at 25 °C and 37 °C. The study employed the use of headspace solid phase micro-extraction (HS-SPME) gas chromatography mass spectrometry (GC-MS), and sensory analysis to track important volatile markers of LO over 15 months of storage. Propanal was used to monitor oxidative changes in n-3 PUFA, with hexanal and pentanal used to monitor changes in powders fortified with n-6 PUFA. Samples stored at 37 °C were found to be less stable than those stored at 25 °C, confirming that storage temperature effects the rate of LO in IMF. Rancid off-flavour was not detected in samples stored at 25 °C until after 15 months. The combination of sensory and volatile analysis provided beneficial information on the oxidative stability of the formulations and concluded that the shelf-life of IMF is dependent on the PUFA content, storage temperature and time.

A study by Clarke et al. [51] found that LO aldehydes and ketones were excessively high in IMF in comparison to WMP and SMP. Overall, painty, oxidised, and rancid attributes were more associated with IMF regardless of storage temperature (21 °C or 37 °C). However, to date very few studies have been undertaken on the impact of LO on the volatile and sensory profiles of IMF. Cesa et al. [62] investigated the effect of storage conditions (20, 28, 40 and 55 °C) on the levels of malondialdehyde (MDA) in IMF and found that the PUFA enriched IMF samples demonstrated good stability at; 20 °C for up to 1 year, 40 °C for up to 3 months, and 55 °C for up to two weeks. A study by Jia et al. [63] investigated the stability of milk based IMF supplemented with PUFA stored at 42 °C and 50 °C for 90 days. The authors found significant differences in colour, possibly due to the Maillard reaction in addition to significant differences in the VOC profile, peroxide value (POV) and headspace oxygen over the storage period.

Most IMF LO studies have only monitored the concentrations of MDA or the POV, with very little research focused on individual VOC associated with LO. Investigating the levels of primary and secondary oxidation products (aldehydes, ketones and alcohols) in IMF in conjunction with sensory analysis will provide more information on the impact of supplementation with PUFA on the rate of LO during storage, the specific VOC involved, and in theory the concentration at which individual VOC begin to adversely influence sensory perception.

### 5.4. Whey Protein Concentrate and Whey Protein Isolate

Whey is used as an ingredient in many food products and generally fractionated to yield products with different compositions and functionalities [64]. WPC has a low fat content of between 3–6.6% *w*/*w*, with negligible amounts of saturated FA, PUFA, and MUFA [65]. As such, LO is not considered a major issue and therefore only limited LO studies of WPC exist.

Tomaino et al. [66] suggested that the starter culture used during cheese production can initiate the oxidation process, which influences the flavour and oxidative stability of liquid whey, ultimately, affecting the characteristics of whey powder. Compared with the control, starter cultures were found to have contributed to the production of acetaldehyde, ethanol, diacetyl, 1-propanol, and 2-propanol. Further results suggested that LO was initiated during the production of the liquid whey and was accelerated during 14 days of refrigerated storage [65].

Jensen et al. [67] studied oxidation in WPC (6.5% *w*/*w* fat) and in whey fat concentrate (WFC) for 12 months at 20 °C. WFC is the remaining fraction of WPC after WPI is removed and has a fat content of 13.5–21.5% *w*/*w*. The study evaluated the primary oxidation products for hydroperoxides, electron spin resonance (ESR) for radicals, secondary volatile LO products by HS-SPME GC-MS, and some protein oxidation products (dimethyl disulfide, benzaldehyde, and dityrosine) by reverse-phase high performance liquid chromatography. WFC was found to be more susceptible to oxidation than WPC, as dimethyl disulfide, benzaldehyde, dityrosine, heptanal, nonenal, hexanal, and radicals were significantly higher, likely due to the higher fat content of WFC.

WPI is produced from WPC and contains minimal fat. Berton-Carabin et al. [68] investigated LO in conjunction with protein oxidation as this is seen as a more prevalent issue in WPI owing to its very high protein content (≥90% *w*/*w*). The authors used controlled levels of LO and protein oxidation to investigate the impacts on the viscoelasticity of whey protein layers at the oil-water interface. Results demonstrated that both protein oxidation and LO led to a decrease in interfacial elasticity when compared to the samples that were not oxidised. LO induced the formation of surface active compounds, which were thought to have formed segregated domains at the interface. Limited studies of LO in WPI suggest that it is not a major issue.

Wright et al. [69] investigated the sensory and volatile stability of WPC80 and WPI stored in polyethylene lidded bins at 21 °C, at 50% relative humidity for 18 months. Sensory properties were evaluated using the descriptive spectrum method while HS-SPME GC-MS was employed to extract and characterise VOC. Differences in the sensory profiles were documented between WPC80 and WPI, in agreement with previous studies on the topic [38,70]. Sixteen VOC were quantified in WPC80 and WPI. The authors chose these VOC as they represented a range of Maillard reaction, LO, or fermentation derived volatiles that were consistently detected in 3 or more whey products. Hexanal was found to be the most abundant compound identified in fresh WPC80, followed by trans-2-nonenal. The study concluded that the flavour of both WPC80 and WPI changed during storage, with increases in the abundance of VOC. The optimum shelf-life for non-agglomerated WPC80 and WPI stored at 21 °C was 12 to 15 months, and 8 to 12 months for steam-agglomerated or lecithin-agglomerated WPC80 and WPI.

## 6. Main Factors Influencing Lipid Oxidation in Dairy Powders

Numerous studies have evaluated the effect of feeding system on the flavour and abundance of VOC in dairy products and bovine diet has been proven to be one of the most significant influencers of VOC and FA profiles of dairy products [71,72,73,74,75]. Milk fat composition can be readily modified by changing a cows’ feeding regimen, but this alteration impacts the protein, urea, citrate and the soluble calcium present in the milk (list not exhaustive). Only a limited number of studies have focused on the effect of feeding system on the sensory, flavour and flavour stability of dairy powders [51,63]. Bovine dietary changes that affect the FA profile of milk are also likely to influence its oxidative stability and flavour [53], but also the stability of resultant powders. Moreover, milk quality and composition are important aspects to consider when producing products such as WMP, SMP and IMF.

The review by Chilliard et al. [76] summarised the effects of bovine forage on milk fat secretion and composition. The study highlighted the need to evaluate how different feeding systems impact on aspects of milk fat quality, such as flavour, oxidative stability and manufacturing value.

Studies investigating the composition of milk produced from many supplemented and altered diets including supplementation with: flaxseed [77], lipid complex (grapeseed oil with synthesised conjugated linoleic acid (CLA) and Atlantic mackerel oil enriched with n-3 FA) [78], iodine [79] marine algae [80], oregano and caraway essential oils [81], hull-less barley [82] and sunflower/fish oil [83] have been undertaken. These studies focused mainly on production performance, milk composition, milk yield, FA composition and to a lesser extent, on flavour and sensory characteristics of the raw, pasteurised or homogenised milk. Volatile analysis was included in most studies but very few combined this with sensory analysis or attempted to correlate both data streams.

Villeneuve et al. [84] investigated 3 types of feeding system (timothy hay, pasture, and silage) and found that untrained sensory panel members could not distinguish a flavour difference between the milk produced from the cows fed hay and the cows fed silage. However, a significant number of panellists could detect a difference between milk from hay-fed cows in comparison to milk from pasture-fed cows. A study by Faulkner et al. [85] demonstrated that feeding system can influence the sensory properties of bovine milk. The flavour compounds from forage can be transferred to milk from the cow through two pathways; by inhalation or digestion, and also through the rumen gases [86]. VOC can also be ingested by the animal and encapsulated in the fat or protein portion of the milk. Pasture based feeding has been attributed to increased herbaceous flavours in milk [85].

A study by Vanbergue et al. [87] investigated the effect of breed (Holstein and Normande), feeding system (high and low energy), and stage of lactation (early, mid and late) on milk fat characteristics in dairy cows. No significant interaction was observed between breed and feeding system. Milk yields were higher for Holstein cows compared with Normande cows throughout lactation and were significantly higher in cows that consumed the high-energy diet. In general, fat content was higher for Holstein cows, but saturated FA were higher in Normande cows and MUFA were higher for Holstein cows. Feeding system had no significant effect on saturated FA content except during early lactation where levels were higher in milk from cows fed the high-energy diet. MUFA and PUFA contents were higher in milk from cows fed grass silage (low energy) vs. corn silage (high energy) during early lactation The study clearly demonstrated the effect of cow breed and feeding system on milk fat characteristics.

It is clear that more comprehensive research is required to establish definitive links between bovine diet and the sensory attributes of subsequent milk and dairy powders, where noticeable changes in VOC and FA profile occur. As well as feeding regime, many other factors influence the sensory attributes of milk and dairy products.

## 7. Impact of Processing Conditions on Dairy Powders

There are several factors involved in milk processing which can affect the stability of the resulting dairy powder and its subsequent sensory characteristics, including preheat treatment [88,89,90,91], and the distribution of fat in the dried powder particles [92].

Baldwin and Ackland [91] studied the effect of 4 preheat treatments (85, 95, 110, and 125 °C) each in combination with 4 holding times (10, 20, 60, and 240 s) on the aroma and flavour characteristics of WMP stored in an air atmosphere at 30 °C for 18 months. Nine sensory characteristics incorporating flavour, aroma, and texture were evaluated by 16 trained panellists throughout storage. Some of the primary sensory attributes associated with WMP were significantly affected by preheat temperature and holding time. Cooked flavour was significantly increased by longer preheat holding time and a higher preheat temperature from 85 °C to 125 °C. Sweetness was higher when longer holding times and low preheat temperature were applied, and at shorter holding times at high preheat temperatures. Oxidised flavour was significantly affected by preheat temperature and holding time. WMP manufactured using short holding times and low temperatures exhibited weak oxidised flavour compared to WMP produced using high heat treatments and longer holding times. Oxidised flavour correlated well with oxidised aroma and was perceivable by panellists after 9 months of storage. The study concluded that preheat temperatures of 95 °C or greater and holding times of 20 s or greater are considered effective in inducing stability against oxidative deterioration. This finding was in agreement with that of Abdalla et al. [57] for NFDM and SMP.

Li et al. [88] evaluated the oxidative stability of milk powders throughout 3 and 6 months of storage at 20 ± 1 °C. Milk powders were stored in plastic bags and stored in a dry, air-tight container. In addition to HS-SPME, POV was used to evaluate the oxidative stability of the milk powders. Milk powders and concentrated milks had higher POV than raw and heated milks. The study also found increased levels of aldehydes and ketonesin stored milk powders when the concentration temperature was 40 °C as opposed to 50 °C. Results also demonstrated that aldehyde and ketone levels in fluid milk, both raw and heated, were lower compared to levels found in concentrated milk and milk powders. The increased number of processing steps involved in milk powder production, such as preheating and spray drying were thought to be the cause of this increase. The temperature of the preheat treatment is important for regulating the technological characteristics of the final product [90].

Stapelfeldt et al. [89] demonstrated that the shelf-life of WMP depends on the preheat treatment of the milk, the temperature at which the WMP is stored and the water activity of the powder. The study compared the storage stability of low-heat, medium-heat, and high-heat milk powders at three water activity values (0.11 a_w_, 0.23/0.17 a_w_, and 0.33/0.31 a_w_), and at two storage temperatures (25 °C and 45 °C). The freshly manufactured milk powder was packed in 400 g cans under a 70% N_2_, 30% CO_2_ gas mixture. The low-heat milk powder (milk pasteurised at 73 °C for 20 s followed by a preheat treatment of 72 °C) had the lowest storage stability as it was subject to severe oxidative changes and non-enzymatic-browning. However, during accelerated storage at 45 °C, the medium-heat (milk pasteurised at 80 °C for 20 s followed by preheat treatment of 72 °C), and high-heat powders (milk pasteurised 88 °C for 20 s followed by preheat treatment of 72 °C) were less susceptible to oxidative changes and enzymatic browning. In the study, thiobarbituric acid reactive substances (TBARS) was used as a measure of sensory quality and values increased to a greater extent in powders stored at 45 °C than at 25 °C.

Park et al. [93] explored the effect of homogenisation pressure on the flavour, and flavour stability of WMP. The sensory properties of the powders were evaluated at 0, 3 and 6 months of storage at 21 °C by descriptive analysis using the spectrum method [94]. The study reported the flavour profiles of WMP produced by various homogenisation treatments were distinct, and that improper or inadequate homogenisation adversely affected shelf-life and flavour stability.

Monitoring the temperature and conditions during the drying of milk is crucial to the overall quality and sensory stability of the end product. Other parameters such as particle size and microbiological stability must also be considered [95].

## 8. Volatile Organic Compounds Associated with Lipid Oxidation in Milk

VOC are a diverse group of carbon-based chemicals with boiling points ranging from 50 to 260 °C [96]. VOC including aldehydes, ketones, alcohols, and ethyl esters represent the primary aromatic constituents of milk. VOC are significant as their quantitative differences can explain the different odours that characterise milk and dairy powders [50,97]. The concentrations of individual VOC in fluid milk are known to affect its sensory properties [85]. GC is capable of identifying >109 molecules of an odour in 1 mL of air, but the human nose has been found to be 10–100 times more sensitive [98]. Therefore, VOC analysis in combination with GC-olfactometry (GC-O) can provide more useful information about which VOC influence sensory perception and the degree of their influence. Although GC-O has some limitations it remains a very useful technique; (1) it can be difficult to identify every odour, as some remain below the limits of detection of the GC-MS, (2) co-elution makes it more difficult to obtain dependable data on those VOC, (3) odours created through interactive effects of two or more VOC cannot be taken into account as the VOC are largely detected as individual compounds, and (4) it is very time consuming and requires extensive panellist training. In addition, some VOC found in dairy products have more than one odour descriptor that may also be dependent upon their concentration as well as the composition of the product [10]. Although the human nose is very sensitive it also has limitations, such as the ‘opinion factor’ of panellists, lack of standards and reproducibility due to differences in capability either related to physical, genetic or health issues [99]. A review by Kilcawley et al. [10] summarises the potentially important compounds in bovine milk and their associated aroma descriptors (Table 1).

In addition to LO, Maillard, and Strecker reaction products, other documented sources of off-flavours in dairy products include the presence of microbial-derived terpenoid compounds, such as endo-borneol, 2-methylisoborneol and α-terpineol [100], sulfur compounds present as a result of heat treatment [101,102], or direct transfer from feed and possibly isoflavone metabolism in the rumen leading to the formation of aromatic phenolic compounds [71,103]. Thus, incorporating GC-O analysis when attempting to identify the source of off-flavours in milk can be extremely beneficial.

**Table 1:** Some potentially important volatiles and their associated aroma descriptors found in dairy products (derived from lipid oxidation).

Compound	Associated Aroma Descriptors	LRI	Odour Reference	LRI Reference
Aldehyde				[71]
Pentanal	Fermented, bready, fruity	735	*	
Propanal	Alcohol, earthy	506	*	
Hexanal	Cardboard like, metallic off flavour, green	837	*	
(E)-2-Nonenal	Green, fatty	1160	*	[104]
Heptanal	Fatty, oily, green, woody	901	*	[71]
(Z)-4-Heptenal	Oily, fatty, green, milky, dairy	901	*	[105]
2,4-Decadienal	Fatty, oily, green, chicken skin-like, fried	1300	*	[106]
Undecanal	Soapy, aldehydic, waxy, floral	1311	*	[107]
Ketone				
Acetone	Earthy, strong fruity, wood pulp, hay	532	[108]	[71]
2-Nonanone	Malty, fruity, hot milk, smoked cheese	1092		[109]
2-Heptanone	Blue cheese, spicy, Roquefort cheese	890		
2-Pentanone	Orange peel, sweet, fruity	727		[71]
3-Octen-2-one	Earth, oily, ketonic, sweet, hay, mushroom-like	1096	*	[51]
2,3-Octanedione	Dill, herbal, buttery	981	*	[110]
1-Octen-3-one	Metallic, mushroom-like	1294	*	[111]
3,5-Octadien-2-one	Mushroom-like, fatty	1030	*	[71]
Alcohol				
1-Heptanol	Sweet, green, woody	972	*	[71]
1-Octanol	Waxy, green, citrus, floral, sweet, fatty, coconut	1116	*	
1-Pentanol	Fermented, sweet, balsam, yeasty, solvent-like	794	*	
1-Hexanol	Green, herbal, alcohol, sweet	894	*	

LRI: Linear retention indices on a DB5 column; * Odour reference from The Good Scents Company [112].

## 9. Qualitative and Quantitative Measurement of Lipid Oxidation Compounds in Dairy Products

There are various techniques and strategies used to measure LO in dairy products. Some commonly used, relatively simple, and practical methods to assess LO are POV, TBARS, and the KREIS test. Their widespread use is mainly due to ease of use and low cost, although they are more qualitative rather than quantitative.

Several analytical methods have been optimised for detecting off-flavours associated with LO in dairy products, such as solvent-assisted flavour evaporation (SAFE), GC-MS [113], and GC-O [114]. GC-flame ionization detection (FID) or GC-MS have become the methods of choice for quantitative VOC analysis. These approaches are undertaken in combination with a specific method to extract and concentrate the VOC using either static or dynamic headspace techniques, sorption-based techniques, liquid based extraction or solvent assisted techniques. As previously mentioned, care must be taken not to increase VOC associated with LO during the analytical technique, as previous studies have demonstrated that certain LO VOC can increase between 37 °C and 60 °C [115], therefore, including appropriate controls is necessary to prevent false positives.

### 9.1. Peroxide Value

POV is still widely used by the food industry as a qualitative indicator of oxidative stability in dairy products as it is inexpensive and relatively easy to use. The titrimetric method is described in the AOAC standard [116], and the spectrophotometric method is described by Østdal et al. [117]. The basis of the assay is a solvent separation, followed by a reaction and absorbance reading at 470–500 nm. However, its accuracy and usefulness is questionable as it only considers the first stage of the LO reaction i.e., the initiation phase [118]. Thus, in theory sensory properties can deteriorate further (due to hydroperoxides breaking down to form odour active oxidation products such as aldehydes, ketones and alcohols) without any increase in POV values. It is also not possible to make a judgement on the sensory characteristics of a product using the POV as hydroperoxides are generally tasteless and flavourless [119]. In addition, the breakdown of hydroperoxides can occur at a faster rate than their formation, therefore it is still possible to have sensory issues at low POV levels. However, the POV can be used as a tentative non-specific, non-quantitative indicator of quality deterioration over time [120].

### 9.2. Thiobarbituric Acid Reactive Substances

TBARS methodology is a relatively simple spectrophotometric assay and remains widely used in the food industry. Some widely acknowledged limitations include a lack of specificity as the method uses the formation of MDA to represent the overall formation of aldehydes, thus providing no information on any individual LO volatiles. In addition, the TBARS reaction is not specific to MDA; the presence of any sugar can react with the thiobarbituric acid and yield a colour change, leading to an overestimation of the extent of LO [121]. The method also fails to account for the numerous aldehydes, ketones and alcohols resulting from LO that are responsible for off-flavours associated with LO in dairy products. Another disadvantage of this method is the requirement for solvents and the associated risk assessments [122]. Studies report the levels of MDA in milk to be between 0.028–0.036 ppm [123], and higher in milk powders (0.3 ppm) [124] and IMF (0.1–1.2 ppm) [125].

### 9.3. KREIS Test

The KREIS test was one of the earliest methods used to determine the oxidative deterioration of vegetable oils and is similar to TBARS in that solvents, a colour change and spectrophotometric measurements are involved. The primary reagent in the KREIS test (phloroglucinol) reacts with aldehydes and ketones to develop a pink colour i.e., a positive result. The KREIS test does not appear to be as widely used as TBARS or POV. A study investigating rancidity in edible oils [126] has linked certain odour-active aldehydes identified using the KREIS test with deterioration in quality and has promoted the use of the KREIS test for the early detection of scission products of FA [127]. The presence of some aldehyde compounds that are not associated with rancidity have been shown to give false positive results and other compounds such as vanillin were shown to interfere with results [128]. Overall, the KREIS test is not considered a reliable LO indicator [129,130].

### 9.4. Physical Evaluation Methods

The application of NMR spectroscopy monitors the change in FA profile in oils by calculating the ratio of aliphatic to olefinic protons. NMR profiling has previously been applied to evaluate variations in the milk metabolite profile [131,132] and more recently has been employed as a tool to identify and determine the quality of the lipid fraction of organic and conventionally produced bovine milk with emphasis on metabolites with potential health benefits [133]. Other physical evaluation methods have been employed to determine the oxidative stability of dairy products, including ESR spectroscopy [33] and evaluating the presence of conjugated dienes present as a result of PUFA oxidation by UV absorption at 234 nm. However, this method was not considered useful for early detection of VOC that cause sensory defects in powders [134]. It is also possible to qualitatively assess the conjugation of PUFA dienes by refractometry [135] and infrared spectroscopy [136].

### 9.5. Analysis of Volatile Organic Compounds by Gas Chromatography

Some of the most commonly utilised volatile extraction methods used in combination with GC to assess LO are outlined below, namely HS-SPME, thermal desorption (TD), SAFE, and sorptive extraction (SE).

HS-SPME has become a standard approach for the volatile profiling of food samples [137]. The basic principle of HS-SPME is that the sample of interest is placed in a sealed vial and heated under controlled conditions so that an equilibrium of the VOC is formed in the headspace which is representative of the sample. However as with all HS techniques, the nature of the sample matrix as well as the chemical properties of the individual VOC have a significant influence on the release of VOC. HS-SPME has become the most widely used volatile extraction technique as it requires minimal sample preparation, is solventless, fully automatable, easy to use, very versatile due to the wide range of fibre phases available, reproducible, and is relatively inexpensive [138]. Once the VOC equilibrium is formed, a polymer phase coated fiber is exposed to the headspace under controlled conditions (time, temperature and agitation). The VOC interact with the phase(s) and the fiber is retracted and subsequently desorbed for 2–3 min in a heated GC injector port (typically between 250–270 °C). The desorbed VOC are transferred onto the GC column in an inert gas flow and separated by their interaction with a GC column phase on heating in a column oven, and subsequently detected and/or quantified either by FID or MS. Thus far, HS-SPME GC-MS techniques have been applied to a variety of dairy products such as raw and pasteurised milk [139,140], dairy powders [141,142,143], and liquid or powdered IMF [144,145]. A review by Merkle et al. [146] summarised the recent developments and applications of HS-SPME for analysis of complex food matrices. The study mentioned the occurrence of the matrix effect i.e., the binding of analytes to the matrix resulting in low concentrations of the analytes in the headspace. Thus, the matrix effect may be an issue when developing a HS-SPME method for the extraction of volatiles from dairy products with increased fat contents. In an effort to off-set the matrix effect, response surface methodology has been used to determine the most useful HS-SPME extraction parameters for the quantification of VOC associated with LO in dairy powders [141].

TD also works on the bases of heating samples to allow VOC reach the gaseous phase. As with HS-SPME, TD is used as an extraction and pre-concentration step prior to analysis by GC. VOC and some semi-VOC are extracted by this technique onto suitable phases packed into TD tubes, with many different phases available that can target individual VOC or chemical classes, or for more generic untargeted approaches. Removal of the trapped compounds from the phase(s) onto the GC column involves heating of the TD tube in a gas flow, and sometimes further concentration is possible using an in-line focusing trap. TD has been applied to a number of dairy products [85,147], milk powder [148], and IMF [149]. A number of application notes are also available on the use of TD [150,151,152]. Its widespread use in dairy products may be limited as moisture management can be problematic.

SAFE is a useful method for the isolation of volatiles from complex food matrices. Engel et al. [153] reported that the application of SAFE to model solutions containing a range of aroma compounds resulted in increased yields from both solvent extracts and fatty matrices (50% fat) when compared with high vacuum transfer. SAFE could be particularly useful for longer chain, fat soluble compounds that have difficulty reaching the gaseous phase. SAFE has been used to prepare volatile extracts from dairy products for GC-O evaluation; Bendall [154] used SAFE to extract volatiles from milk to be analysed via GC-O, 71 different aroma-active compounds were isolated from the milk, 66 of which were identified. However, SAFE has limitations, such as tedious sample preparation, the requirement for solvents, requirement of expensive specialist glassware, reproducibility issues, manual sample manipulations, and may require further concentration steps prior to introduction to the GC [155].

Stir bar sorptive extraction (SBSE) is a solventless technique with simple sample preparation that has been used for flavour research of dairy products including dried dairy ingredients, and milk. Traditionally this technique employed glass-encapsulated magnetic bars with a sorbent coating to extract volatiles. Stir bars can be immersed within a liquid sample or suspended in the headspace of a solid, liquid, or gaseous sample during the extraction process. Volatiles are typically thermally desorbed followed by a cryofocusing step and GC-MS [156,157,158]. Baltussen et al. [156] was one of the first to describe this technique, and employed a polydimethylsiloxane (PDMS) phase to extract and concentrate the VOC. Park and Drake [159] used SBSE for the extraction of flavour compounds from NFDM concentrated by reverse osmosis or evaporation and found that the volatile profiles were consistent with the descriptive sensory results.

Faulkner et al. [85] achieved good results for milk samples (up to 65 volatile compounds from a range of chemical classes) using a new high capacity SE technique called HiSorb followed by GC-MS analysis. Currently PDMS-coated stir-bars are the only phase commercially available for these techniques, which somewhat reduces the applicability of SBSE to the extraction of non-polar compounds due to the poor extractability of more polar analytes [157]. However, Ochiai et al. [160] demonstrated that solvent-assisted SBSE improves peak resolution and extraction efficiency of polar and non-polar compounds. Moreover, Schiano et al. [161] concluded that solvent-assisted SBSE provided the most consistent detection of selected compounds in commercial milks, although the levels of compounds detected were not significantly (*p* > 0.05) higher compared to conventional SBSE or SPME extraction methods. Some of the most common techniques used for the extraction of volatiles from dairy powders are outlined in Table 2.

### 9.6. Gas Chromatography Olfactometry

As previously mentioned, the advantage of including GC-O analysis is that it allows trained human assessors to identify VOC that are aroma-active and thus contributing to sensory perception in real time [168]. This enables VOC that are contributing to the overall flavour to be identified and even their potential sensory influence in terms of intensity and character to be defined. The integration of GC-O and GC-MS and/or GC-FID techniques also makes it possible to establish direct relationships between a compound present in a food sample and any correlated odour. However, it is important to note that odours are extremely complex mixtures often consisting of numerous VOC which vary in concentration. VOC can interact synergistically or additively to produce the overall odour of a product [169]. Therefore, while it is beneficial to know which VOC in a sample are odour active, the overall odour of a product can differ from that of each individual VOC. Friedrich and Acree [170] and Rychlik and Bosset [171] provided good descriptors of VOC present in dairy products as detected by GC-O. Kobayashi and Nishimura [172] employed thirteen panellists to compare WMP samples from different regions using GC-O analysis, and concluded that the differences between the WMP based on region was caused by differences in the balance of the aroma-active VOC present.

A limited amount of studies have included GC-O analysis for the VOC in SMP samples. Karagül-Yüceer et al. [166] undertook GC-O analysis on six NFDM powders, a product that is consumed directly as well as being used as an ingredient in other preparations. The study identified a wide range of aldehydes, ketones and free FA which were found to be responsible for the generation of flavours in NFDM over storage. Samples were analysed by GC-MS, GC-O, and sensory analysis. Methional, a Strecker degradation product of methionine, was identified as an off-flavour compound with its corresponding aroma being characterised as boiled potato-like [173,174]. These methods ensured a comprehensive evaluation of the products sensory characteristics in terms of the main source VOC responsible. This study also verifies the importance of raw milk quality even as an ingredient in end product applications.

## 10. Sensory Analysis

Regardless of the processing dairy products undergo, consumer acceptance remains primarily based on appearance and flavour [175]. As milk has a naturally subtle and somewhat bland flavour, any development of off-flavours is relatively easily perceived by the consumer [58]. The impact of different feeding systems, production regions, cultural differences and storage conditions have been identified as the motivating factors behind dairy product purchase [72,73,85]. Consumers have a desire to know more about where their milk and milk products are coming from and how they are produced [176]. Previous sensory studies have employed between 25 and 100 panellists for consumer testing and this technique has been widely applied to dairy products [177,178,179]. Full descriptive sensory analysis requires fewer panellists as they are trained on how to specifically assess the product using pre-defined sensory attributes [52]. Panellists are not asked about their own liking or preferences toward the product, but rather they are employed as calibrated analytical instruments to give results on the intensity of particular descriptors known to be characteristic to that product. When recruiting a panel for sensory analysis, screening tests are performed to ensure each panellist provides an accurate and reliable result. Selection factors include; continued availability, health status, ability to perceive flavours, familiarity of the product under evaluation, previous experience, allergies, and medication. However, even with a stringent selection protocol, the ‘opinion factor’ continues to play a role in sensory analysis due to genetic and cultural differences between panellists [180].

When implementing quantitative descriptive sensory analysis, efforts should be made to ensure the sampling technique is consistent across the panel and each panellist understands how the odours and flavours are to be interpreted and described. Selecting the product descriptors (lexicons/attributes) in the final evaluation is generally a consensus process, decided upon in a focus group, conducted prior to sensory evaluation [181]. The final descriptors must comprehensively describe the sensory attributes and their intensities.

Combining sensory analysis with any of the aforementioned GC techniques provides more detailed information on numerous aspects of dairy products including consumer acceptability, levels of rancidity, extent of LO, and intensities of compounds with known odour descriptors. Moreover, the concentration of LO compounds perceived as unacceptable can be determined. The review by Kilcawley et al. [10] summarises recent studies that included sensory analysis of dairy products and the various sensory methodologies used.

Table 1 summarises the aromas associated with some important volatile compounds in milk. LO generally results in off-aromas and flavours in dairy powders including painty (hexanal, nonanal) [6], cardboard-like (hexanal, pentanal) [6,8], metallic (hexanal, pentanal, vinyl ketones) [182], and fishy (carbonyl compounds, 2,4-unsaturated aldehydes, trimethylamine) [182]. Some studies have suggested that producing dairy powders below 4% *w*/*w* moisture can delay the development of fishy and tallow off-flavours [183], however it is most likely also dependent upon the range of factors that are known to influence the concentration of the various VOC responsible. Sulfur compounds can be problematic as they have very low odour thresholds and are as associated with cooked flavours in ultra-heat-treated milk [184].

Jo et al. [185] documented an interaction between milk proteins and sulfur compounds in milk, affected by serum proteins associated with casein during heat treatment. The study confirmed that hydrogen sulfide and carbon disulfide contributed to eggy and sulfur/burnt flavours in heat-treated milk, respectively. Interestingly, dimethyl sulfide, dimethyl disulfide, dimethyl trisulfide, dimethyl sulfoxide, and methional were found not to be associated with sulfur/burnt and eggy flavours in heat-treated milk.

Various studies have investigated the impact of oxidation on the flavour and stability of dairy products [6,50,69,93,186]. A review by Su et al. [187] summarises the sensory lexicons used for the evaluation of dairy products and established the connection between off-aroma lexicons and volatile formation pathways existing in dairy ingredients. This review concluded that many off-aromas are as a result of protein, fat, and sugar breakdown products from lipid degradation and Maillard reaction pathways. The review suggested that to minimise off-aromas developing in dairy products, in particular high protein formulations, a high quality starting material is required, and processing parameters should be monitored and adjusted accordingly to decrease the rate of flavour degradation.

## 11. Conclusions

There are numerous factors that influence the rate of LO in dairy products; such as cow breed and diet, stage of lactation, levels of PUFA and unsaturated FA, storage and processing conditions (exposure to heat, oxygen and/or light). Processing conditions require monitoring to ensure the quality of the end product is consistent and free from any undesirable flavours. The quality of the raw milk used for the production of dairy powders is very important, but also the manner in which the milk is handled, processed and stored has a significant impact on the extent to which the milk fat is oxidised throughout its shelf-life. LO has been shown to impact on the quality, nutritional and sensory properties of various dairy powders resulting in undesirable flavours and reduced shelf-life. Many analytical techniques are available for the qualitative and quantitative analysis of LO in dairy powders, many of which can be used in combination to elucidate more detailed information. This is especially the case where sophisticated techniques such as GC-MS can be used to identify and quantify individual VOC associated with LO, but in combination with GC-O and/or sensory analysis can provide a much more in-depth understanding of the whole LO process pertaining to a specific product. Therefore, using this approach to determine the concentrations of VOC associated with LO that adversely impact sensory perception, can provide insights into the production parameters that could maximise shelf-life and product quality of dairy powders.

## Figures and Tables

**Table 2:** Summary of the primary methodologies used for volatile extraction and analysis of dairy powders.

Method	Advantages	Limitations	Applications	Reference
**Extraction Methodology**				
Headspace solid-phase microextraction (HS-SPME)	Minimal sample preparation Does not require organic solvents Simple to use ‘Clean’ method in comparison to LC High sample throughput Reproducibility Large selection of phases	Fiber saturation Low phase capacity Possible carryover of compounds	Wide range of volatiles in food products Raw and pasteruised milk Liquid and powdered infant formulas Milk powders	[137,138,139,141,142,144]
In-tube extraction (ITEX)	Does not require the use of solvents Dynamic extraction Well matched to the analysis of trace organic compounds	Repeatability issues Possible issues with moisture and needle blockage	Volatile organic hydrocarbons from aqueous samples	[162]
Thermal desorption (TD)	Good sample throughput Minimal sample preparation Does not require organic solvents Large selection of phases available Sample collection and enrichment capabilities	Tedious if not automated Moisture control	Bovine milk Milk and cheese Milk powder	[85,147,148,150,151,152]
Solvent-assisted flavour evaporation (SAFE)	Simple method Capable of rapid and in situ identification of volatile compounds	Requirement for solvents Expensive glassware Requirement for risk assessment	Milk Skim milk powder (SMP)	[154]
Stir bar sorptive extraction (SBSE)	High effectiveness for the extraction of non-polar and medium-polarity compounds Large amount of phase Good sensitivity and recovery Automated systems under development	Manual removal and washing of stir bar required if not automated	Liquid samples or liquid extracts Dairy products Can be used for headspace analysis	[151,159,160,163]
HiSorb extraction	Effective for the extraction of volatile and semi-volatile compounds Large amount of phase Possible to perform immersive and headspace extraction Automated systems available	Extended extraction times One phase currently available	Liquid samples or liquid extracts Can be used for headspace analysis	[164]
**Identification Methodology**				
Mass Spectrometry (MS)	Powerful compound identification abilities Can compare spectra to libraries Useful for unknown analysis Qualitative analysis Versatility	Reproducibility for quantification purposes	Various volatile and semi-volatile dairy and food products	[51,71,86]
Flame Ionised Detector (FID)	Reproducibility Sensitivity Reliability Quantification abilities	Requires standards for identification No identification ability	Various volatile and semi-volatile dairy and food products	[165]
Gas chromatography olfactometry (GC-O)	Ability to link volatile organic compounds to odour descriptors Provides good odour descriptors Allows for odour thresholds to be determined	Time consuming Ongoing requirement for panel members Must be coupled with the correct extraction method—possible method development required	SMP Any food sample with odour above threshold level	[166]
GCxCG-ToF-MS (Time of Flight-MS)	Good for the separation of complex mixtures Generation of 3D plots Good sensitivity Enhanced resolution Ability to separate co-eluting peaks in the second dimension Ability to reduce or enhance elements of the chromatogram in the second dimension	Complexity of the data generated	Milk lipids	[167]
